# Cats, dogs, and sticky worms: invasion by land flatworms (Geoplanidae) is facilitated by household pets

**DOI:** 10.7717/peerj.20713

**Published:** 2026-02-10

**Authors:** Jean-Lou Justine, Leigh Winsor

**Affiliations:** 1 ISYEB—Institut de Systématique, Évolution, Biodiversité, Muséum National d’Histoire Naturelle, Paris, France; 2 James Cook University, Townsville, Queensland, Australia

**Keywords:** Citizen science, Pets, Dogs, Cats, Invasive Alien Species, Invasion Biology, Flatworms

## Abstract

**Background:**

It is well known that the main vector of invasion by land flatworms has been the export of potted plants from their countries of origin to the invaded country. Within the invaded country, transport to garden centres where the plants are sold, and transport to the buyer’s garden, are also carried out by humans. However, it is less clear how flatworms can then invade neighbouring gardens, given their slow movement rates.

**Methods:**

We re-examined citizen science reports in metropolitan France received over more than 12 years (2013–2025), searching the 6500 original emails for keywords suggesting transport by pets.

**Results:**

We found 15 citizen science observations of cats (13) or dogs (2) with flatworms stuck to their fur. Surprisingly, all reports concerned the species *Caenoplana variegata*, the two-tone planarian, even though this species is not the most abundant in gardens in France. Over the period 2020–2024, observations of *C. variegata* on dogs and cats represented 7.3% (10/137) of reports.

**Discussion:**

We suspect that transport by domestic animals is a significant factor favouring invasion by *C. variegata*, which possesses a particularly sticky mucus adapted to arthropod predation. This is compounded by the fact that the species reproduces asexually in Europe, and therefore the transport of a single individual may be sufficient to facilitate an invasion. We calculated a conservative estimate of the distances travelled outdoors by all the dogs and cats in France, which was 18 billion km/year; if only a tiny proportion of these journeys involve pets carrying flatworms, this transport as a dispersal factor becomes entirely plausible.

**Conclusions:**

We believe that animal transport is a significant factor favouring land flatworm invasion, but that this does not apply to all species. A citizen science initiative could provide a better understanding of the extent and importance of animal transport as a factor for land flatworm invasions in other countries.

## Introduction

Invasive species are considered a major threat to biodiversity and cause significant economic losses (Haubrock et al., 2021). Among these invasive species, terrestrial flatworms (Platyhelminthes, Geoplanidae) are the subject of a growing number of literature articles (De Waart et al., 2025; Justine, Gastineau & Winsor, 2024; Justine et al., 2018; Justine et al., 2020; Mori et al., 2022a), despite the small total number of known species, which is less than 1,000 (Sluys, 2016). In Europe, three terrestrial flatworm species were recently added to the list of invasive alien species of Union concern, in July 2025 (European Union, 2025). The pathway for the introduction of invasive flatworms from their native land to the invaded country is well known: import of potted plants (Sluys, 2016). Flatworms are able to survive for weeks in or under a pot containing plants without eating. The steps by which the worms then invade an entire country from a few plant shipments are less well understood. Most likely, imported plants are first stored in garden centres, where the worms encounter optimal conditions intended for plants, but also perfectly suited to flatworms: misting during the summer, which protects them from drought; and protection against frost in the winter, which prevents them from facing the cold. Flatworms are thought to proliferate en masse in these garden centres. Amateur gardeners then come to the garden centre, buy potted plants, bring them home, and thus infest their garden with the flatworm. All of these steps are under the responsibility of individuals and take various mechanised routes (plane, boat, truck, or car), which leads to rapid dispersal.

The final step is less well understood, *i.e.,* how worms invade neighbouring gardens from an infested garden. While this step is clearly simple for a flying invasive species, such as the emblematic Asian hornet, it is more mysterious for flatworms. The movement of the worms themselves can only very partially contribute to the invasion, since the species move slowly on the ground. Plant exchanges between neighbours are a possibility (Sluys, 2016), but are probably only rare and isolated events that cannot explain the invasion of an entire municipality.

Our attention was drawn to observations of flatworms attached to the fur of domestic animals in the reports we received from citizen science. In this article, we attempt to quantify the importance of this transport by animals, based on the abundant available data from citizen science on invasive flatworms in metropolitan France. To our surprise, we quickly found that only a single species was cited in these reports, which is the two-tone planarian, *Caenoplana variegata* (Fletcher & Hamilton, 1888). In this article, we hypothesise that transport by cats and dogs significantly contributes to the final stage of invasion for this species. We also examine the possibility that this transport of an invasive alien species by animals occurs with other species of flatworms.

## Materials and Methods

### Origin of data

The origin of the data used here deserves a detailed description. Since 2013, one of us (JLJ) has supported a citizen science programme on invasive flatworms in France. The data used here come from several sources, in chronological order: (1) from 2013 to mid-2023, data were received in the form of emails sent by individuals, accompanied by photographs. These observations were all compiled by staff at the Institut National du Patrimoine Naturel (INPN), particularly Jessica Thévenot, in a database publicly available by the INPN (https://inpn.mnhn.fr/espece/jeudonnees/2107, currently not functional); (2) from mid-2023 to mid-2024, observations received by email were sent to the INPN and have not yet been compiled in a database; (3) from mid-2024, it was decided that compiling the information sent by individuals into a database was too costly in terms of time and labour and JLJ therefore sent, in response to each report, an email inviting people to enter the data directly into the INPN databases *via* a smartphone application, “INPN Espèces” (https://inpn.mnhn.fr/accueil/participer/inpn-especes, currently not functional). The photographs received *via* this application are also validated by JLJ, directly in the INPN dedicated website (https://determinobs.fr/#/home, currently not functional). After validation, the information is anonymised, with the names of the people not known, and any free-text comments entered into the application by the user not visible. The dataset is freely available from the Global Biodiversity Information Facility (GBIF) (https://doi.org/10.15468/p2y2z6). It should also be noted that in parallel with the periods when reports were received by email, some people spontaneously sent information *via* the INPN Espèces application. Similarly, reports received in the public database include some older reports, submitted between 2013 and 2023 by individuals who sent photographs taken earlier (from 1999 for the earliest case).

For this work, we used all available data and systematically returned to the observers’ original emails to verify each piece of information and avoid any possible errors that occurred when entering the raw data into the databases. All emails were retained. A total of 6,500 emails were examined; this figure is higher than the number of platyhelminth observations for several reasons: (1) it includes irrelevant reports (earthworms, leeches, and various animals); (2) it includes reports without photographs, which were not considered; and (3) it sometimes took several email exchanges to obtain information or additional photographs. We searched these emails for the following keywords (in French): “cat”, “dog”, and a few words referring to clothing, such as “shoes” or “trousers”. Irrelevant observations, such as worms found “attached to the dog’s bowl”, were eliminated. We considered only cases where reports clearly stated that worms had been carried by their pets.

### Map

We used data freely available in the Openobs database (https://doi.org/10.15468/p2y2z6). Openobs (https://openobs.mnhn.fr/, currently not functional) offered tools for exporting maps. Data on *C. variegata* in Metropolitan France and Corsica included 447 observations (up to 5 May 2025), mainly from citizen science observations individually verified by one of us (JLJ) over the past 12 years. Records from pets were manually added to the map.

## Results

We found a total of 15 citizen science observations (Table 1) of invasive flatworms being transported by cats (13 observations) and dogs (two observations). Additionally, we found a single report of a flatworm being transported attached to the hem of a pair of trousers. In all cases, the only species of flatworm involved, identified by photograph, was *C. variegata*. For cats, two observations specified that the cats were long-haired (one referred to as “long-haired”, the other as “Persian cat”).

**Table 1:** Observations of flatworms in France with mentions of cats and dogs in the comments from the observer. All records were verified from original emails and photographs. There are 15 observations, *i.e.* 13 on cats and 2 on dogs. All observations are of *Caenoplana variegata*. For the period 2020–2024: 10 observations, 8 on cats and 2 on dogs.

#	Database	Date	Commune	Department	Details (in original French)	Details (translated into English)	Pet
**01**	Public	2010-05-25	Anglet	Pyrénées-Atlantiques	“il est [...] très difficile de le retirer d’un bec d’oiseau ou des poils du chat qui nous en rentre dans la maison au moins 2 fois par an”	“It is [...] very difficult to remove it from a bird’s beak or from the hair of the cat that gets into our house at least twice a year”	cat
**02**	Public	2018-10-11	Antibes	Alpes-Maritimes	“j’ai vu ce vers de terre curieux sur le carrelage, je pense que c’est mon chat qui l’a transporté par ses poils”	“I saw this strange earthworm on the tiles, I think it was my cat that carried it on his fur”	cat
**03**	Public	2019-09-27	Saint-Maur-des-Fossés	Val-de-Marne	“sur les poils du ventre de mon chat, (qui se ballade dehors car j’habite au rez-de-chaussée)”	“on the hairs on my cat’s belly (that wanders outside because I live on the ground floor)”	cat
**04**	Public	2022-10-08	Marseille	Bouches-du-Rhône	“trouvé dans les poils de ma chienne après une promenade dans une pinède”	“found in my dog’s hair after a walk in a pine forest”	dog
**05**	Public	2022-10-23	Mouans-Sartoux	Alpes-Maritimes	“sur la chaise de la salle à manger ou dormait ma chatte”	“on the dining room chair where my cat slept”	cat
**06**	Public	2022-11-01	Montfavet	Vaucluse	”dans les poils d’un de nos chats”	”in the hair of one of our cats”	cat
**07** ^*^	Email	2024-07-10	Nantes	Loire-Atlantique	“sous la patte de ma petite chienne”	“under the paw of my little dog”	dog
**08**	Email	2024-07-16	Carquefou	Loire-Atlantique	“trouvé sur un chat fréquentant les jardins”	“found on a cat frequenting the gardens”	cat
**09**	Email	2024-07-22	Bléré	Indre-et-Loire	“ver collé aux poils de mon chat qui rentrait du jardin”	“worm stuck to the hair of my cat who came back from the garden”	cat
**10**	Email	2024-08-04	Loos	Nord	“ramené à l’intérieur par nos chats” “nous avons un petit jardinet”	“brought inside by our cats” “we have a small garden”	cat
**11** ^*^	Email	2024-08-26	Saint-Jean-de-Marsacq	Loire-Atlantique	“enroulé dans les poils de mon chat”	“wrapped in my cat’s hair”	cat
**12** ^*^	Email	2024-08-27	Grandchamps-des-Fontaines	Loire-Atlantique	“dans la gamelle d’eau intérieure de mon chat”; “j’en ai retrouvé un sur mon chat à poils long”	“in my cat’s indoor water bowl”; “I found one on my long-haired cat”	cat
**13** ^*^	Email	2024-10-07	Saint Herblain	Loire-Atlantique	“dans les poils de notre chat qui revenait du jardin”	“in the hairs of our cat that was coming back from the garden”	cat
**14**	Email	2025-01-22	Hostens	Gironde	“Ils s’accrochent aux poils de mes chats persans”	“They cling to the hair of my Persian cats”	cat
**15**	Email	2025-03-02	Villeneuve-sur-Lot	Lot-et-Garonne	“En rentrant mon chat était tout bizarre et j’ai vu un truc bouger et s’enrouler a sa patte. [Je l’ai] enlevé avec une pince et il était très gluant, collé dans les poils.”	“When it got home my cat was acting all weird and I saw something moving and wrapping itself around his paw. [I] removed it with tweezers and it was very slimy, stuck in the fur.”	cat

**Notes.**

* Records illustrated by photographs in Fig. 2.

We aimed to determine what proportion of the observations were related to reports on domestic animals. Given the complexity of the successive stages of data collection (see Materials and Methods), and the fact that one observation, obtained in 2014, had actually been made in 2010, it was not possible to accurately count the total number of observations of flatworms over the entire duration of the observations 2010–2025. However, we were able to accurately count the total number of observations over the period 2020–2024 (Table 2) from the available data in the determinobs.fr database: 137 observations of *C. variegata*. Observations involving cats (8) and dogs (2), for a total of 10 over the five-year period, represent 7.3% (10/137) of all records concerning *C. variegata*. Table 2 also indicates that this species, the only one reported in association with cats and dogs, is not the most frequently observed in metropolitan France, as *Obama nungara* Carbayo et al., 2016 is recorded more often (233 *vs.* 137 observations). However, no reports of flatworms found on animals correspond to this second species.

**Table 2:** Observations of Geoplanidae species from citizen science and mentions of transport by cats and dogs. We compared the number of observations of transport in *Caenoplana variegata vs* those in Obama nungara, which is the most commonly recorded species. Based on the Openobs website, publicly available at: (https://openobs.mnhn.fr/). Period limited to 2020–2024 (5 years).

Species	Total observations	Observations with cats, dogs	Percentage
*Obama nungara*	233	0	0%
*Caenoplana variegata*	137	10	7.3%

**Figure 1:**
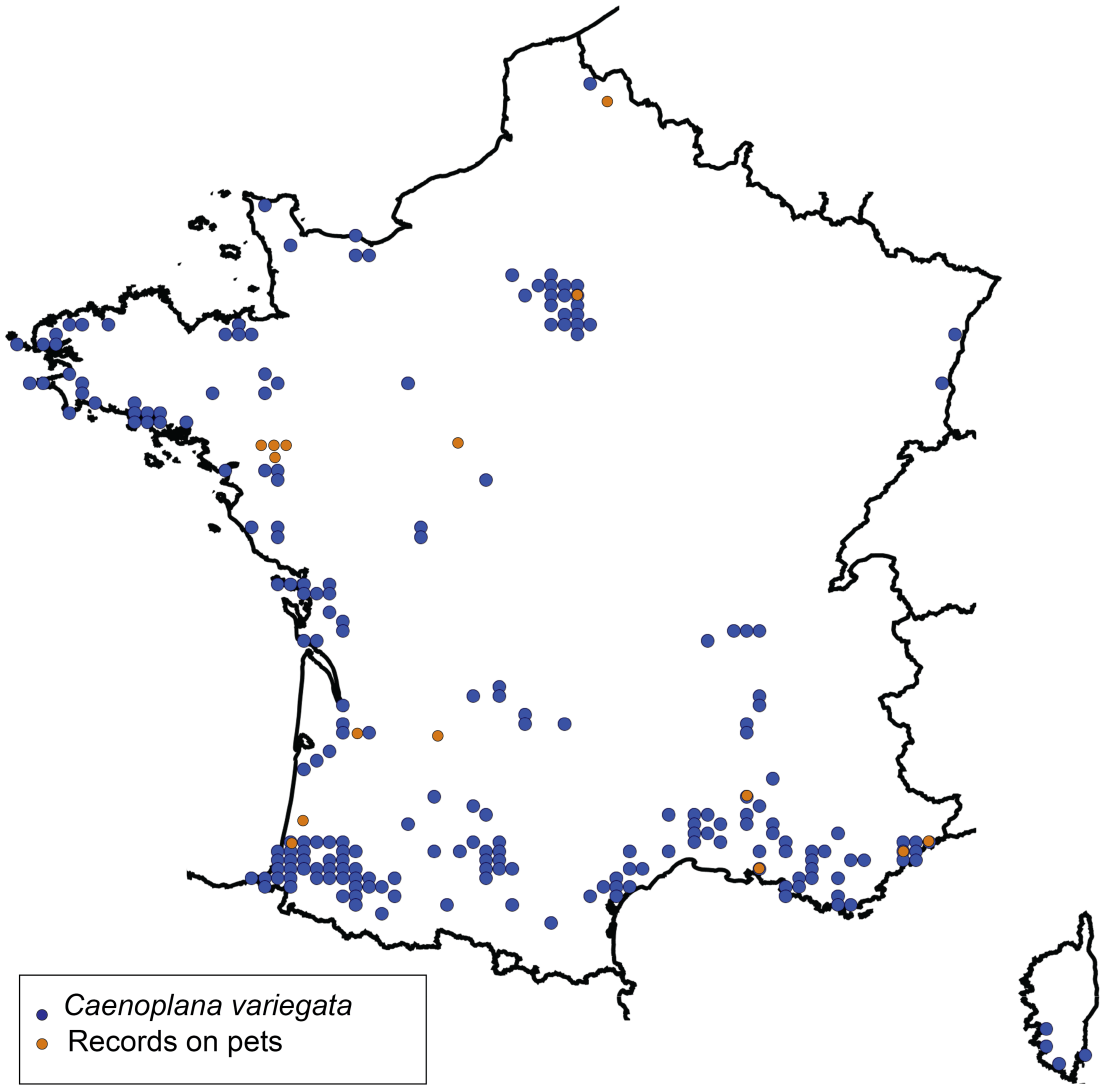
Map showing the distribution of the two-tone planarian, *Caenoplana variegata*, in metropolitan France. The map is based on 447 observations recorded between 2010 and 2025 in the OpenObs database (https://doi.org/10.15468/p2y2z6), each individually verified by one of us (JLJ). The map was generated using OpenObs (https://openobs.mnhn.fr/, currently non-functional). Observations of planarians on domestic pets, which are the focus of this study, are also indicated.

**Figure 2:**
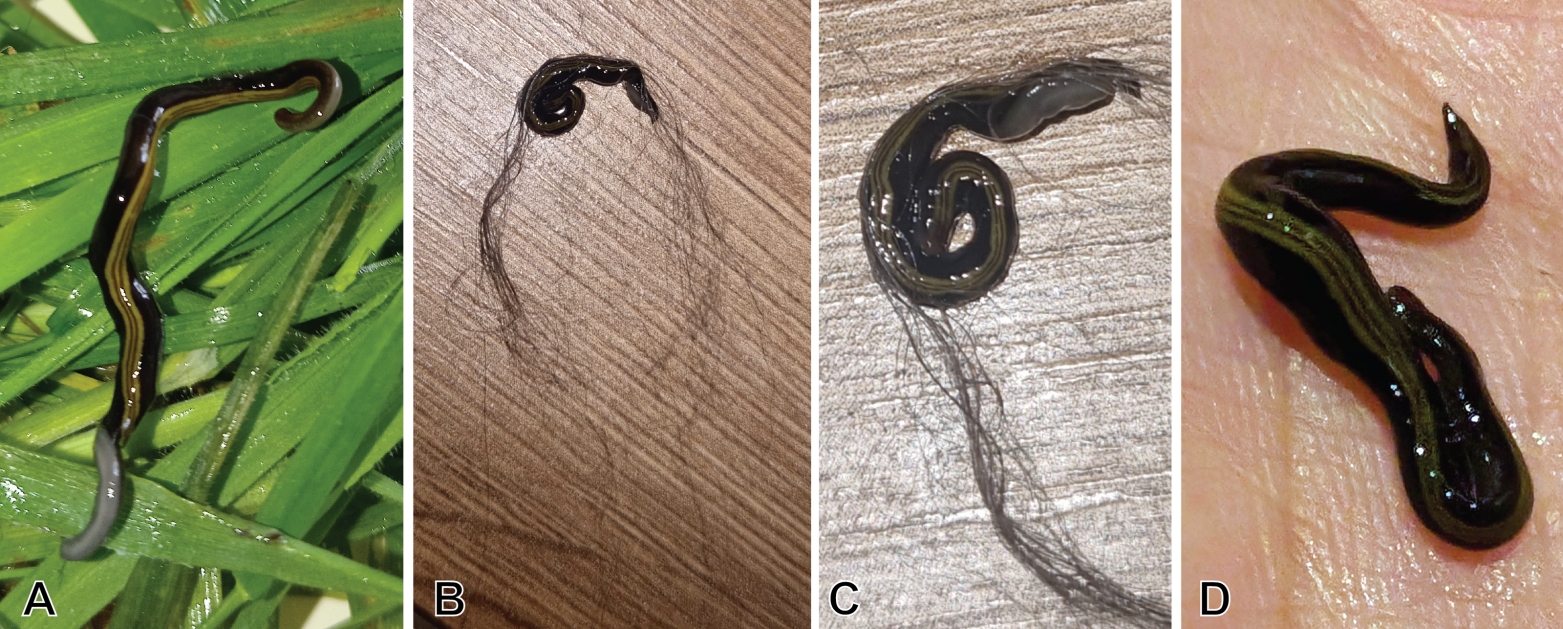
Example of photographs, received *via* citizen science, showing *Caenoplana variegata* collected from domestic pets. (A) Collected from a dog, (B–D) collected from cats. Cat hair still attached to the flatworm is visible in B and C. A, Case #07 in Table 1; B, Case #11; C, Case #12; D, Case #13. A, photograph by Catherine Vaillant; B, C, photographs by anonymous contributors; C, photograph by Floriane Delaval. Anonymous contributors signed a copyright consent for their photographs, but did not wish to have their name included in a publication.

**Figure 3:**
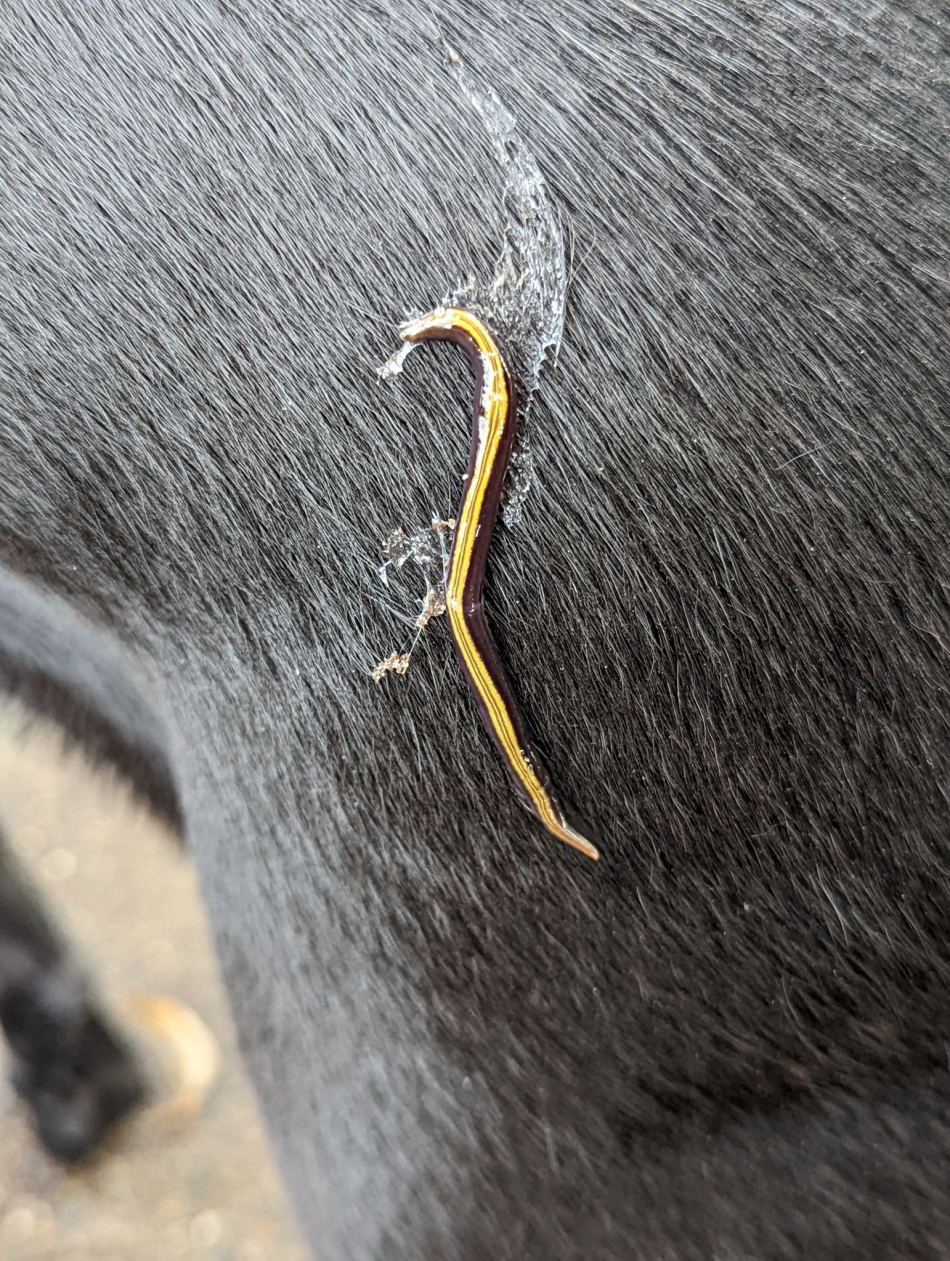
Photograph of a two-tone planarian, *Caenoplana variegata* on a domestic pet’s fur. The finding at Sydney University, New South Wales, Australia, was recorded in iNaturalist as “found on my dog’s butt”. This observation corresponds to #13 in Table 3. Photograph by Dr. Rosie Steinberg, CC BY 4.0, from iNaturalist https://www.inaturalist.org/observations/109693279.

**Figure 4:**
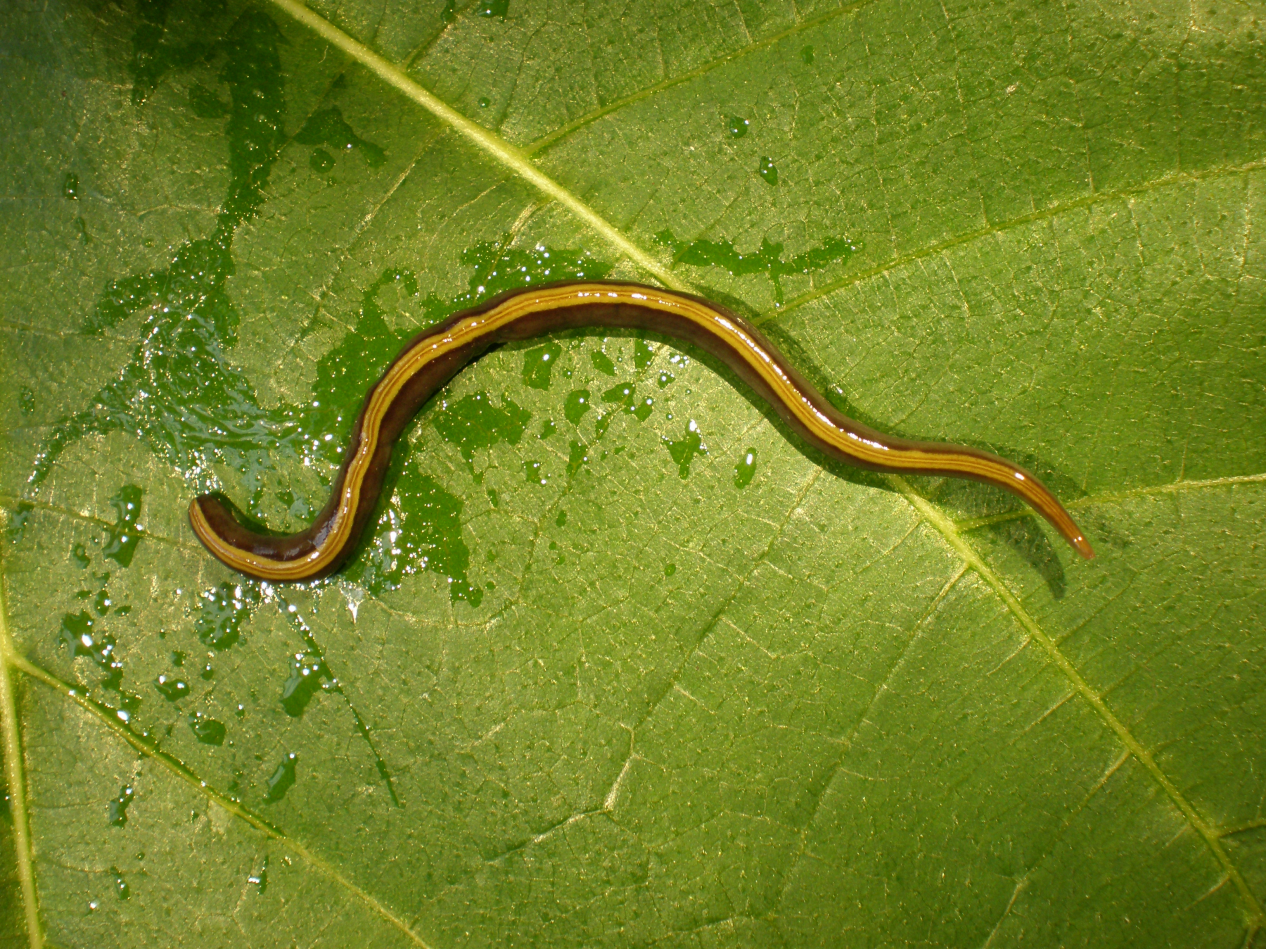
The two-tone planarian, *Caenoplana variegata*. Photographs of a living specimen, now registered in the MNHN collection as MNHN JL144. This specimen was used for a detailed molecular account (Gastineau et al., 2024). Specimen placed on a leaf. Head is right. Unscaled, but the size of the specimen was about seven cm in length. Photograph by Jean-Lou Justine.

Figure 1 presents a map of the overall distribution of *C. variegata* in metropolitan France, based on 447 observations collected between 2010 and 2025. The figure also includes observations from domestic pets, which are the focus of the present study. No distinct geographic pattern was observed for these records, which occur across all regions where the species is present, except for a slight overrepresentation in the Loire-Atlantique department, likely due to chance. Photographs sent by individuals show, in several cases, worms tangled in cat hair or stuck to dog fur. For a few photographs, we were able to obtain permission to publish; these are shown in Fig. 2. A photograph from iNaturalist, taken in Australia, is shown in Fig. 3. The general appearance of living *C. variegata* is shown in Fig. 4.

## Discussion

### Speed of movement and invasion of Geoplanidae

When it comes to speed, for an invasive species, two concepts must be clearly distinguished: (a) how fast an individual moves physically, and (b) how fast the species is able to invade an area following the arrival of an individual (or a breeding pair in the case of sexual species).

Concerning the first point, geoplanids are relatively slow animals, moving along the ground using vibratile cilia located on the sole on the underside of their body, made possible by the presence of mucus covering the entire body, and by undulations of the ventral muscles. Speeds reported in the literature for various species were 1 to five cm min^−1^ (Jones, 1978; Pantin, 1950), and 0.3 mm.s^−1^ (= 18 cm.min^−1^) for *Platydemus manokwari* (Gerlach, 2019). For the species *C. variegata*, our (unpublished) observations are of the order of a cm per minute (ca. one cm.min^−1^). If we imagine, theoretically, a worm moving non-stop in a straight line, at maximum speed, for one night, *i.e.,* 8 h, it could travel 0.48 m, and if moving in this way all year, 175 m/year.

Little is known about the speed of invasion of an area. For *P. manokwari*, two figures are conventionally reported: the worms took one year to invade two garden areas separated by 30 m of lawn (Winsor, 1990), and they eliminated *Achatina* snails within a 180 m radius around the point of introduction after one year (Muniappan, 1987). For *Kontikia andersoni* Jones, 1981 on a subantarctic island, recent observations have suggested an invasion rate of 500 m per year (Houghton, Terauds & Shaw, 2022). These invasion rates are comparable with what the theoretical calculation of “straight-line movement at maximum speed” above would suggest.

### Significance of the observations

Our work primarily concerns observations in metropolitan France. The main reason is that citizen science data for this geographic region are substantial and well-verified scientifically. De Waart et al. (2025) showed that, for example, for the species *Obama nungara*, the number of observations in France is higher than in all other countries. This is also true for *C. variegata*.

The question that we consider the most important to answer, and which motivated our work, is whether these reports of carriage of land flatworms by domestic pets are anecdotal, or reflect a real biological phenomenon.

We would have tended to answer in the negative if the proportion of land flatworms found on dogs and cats had been the same for all flatworm species. On the contrary, our observations in France show that only one species, *C. variegata*, is affected, even though this species is not the most abundant, either in terms of the number of locations or the number of individuals (Table 2). In comparison, *Obama nungara* is much more widespread in the country, and the number of individuals in a single garden is often very high, reaching hundreds or even thousands (Justine et al., 2020; Noël et al., 2025). However, not a single observation of animal transport was reported for this species. Fisher’s exact test yielded a *p*-value of approximately 3.9 × 10^−^^5^, indicating a highly significant difference between the proportions of *C. variegata* (10/137) and *O. nungara* (0/233) observed on animals. We therefore consider the observations of the transport of *C. variegata* individuals by domestic animals to be significant.

We also note that our citizen science data only report the observation of the presence of worms when pets returned home. Cases where a worm attached itself and then fell off during the pet’s journey, before the pet returned to the starting point, are therefore not counted. Also, a number of citizen science flatworm reports are often quite surprising because the worms are found in rooms inside houses with no direct communication with the outside. It is possible that this also corresponds to worms brought inside by domestic pets that were not noticed by their owners upon arrival in the house; we have not attempted to count these observations. However, this suggests that the number of observations of transport by domestic pets is underestimated in this study.

The single observation of a worm attached to a pair of trousers also suggests that transport by humans does occur, but that it is numerically less significant than that by pets. Only one species of flatworm was involved, again *C. variegata*.

### Potential reasons why *C. variegata* was the only species involved

*Caenoplana variegata* is native to Australia and has now been introduced to many European countries (De Waart, 2016; de Waart, Thunnissen & Sluys, 2021; Dorigo et al., 2020; Gastineau et al., 2024; Ghezzi, 2024; Glaw et al., 2024; Jones et al., 2020; Justine, Gastineau & Winsor, 2024; Justine, Thévenot & Winsor, 2014; Mori, Magoga & Mazza, 2022b; Mori et al., 2023; Vardinoyannis & Alexandrakis, 2019). A recent record (2024) in iNaturalist shows that the species has also arrived in North Africa, in Morocco (https://www.inaturalist.org/observations/235058668).

*Caenoplana variegata* is a species with an elongated and slender body; its total length can reach 170 mm when extended, with a width of five mm. The species is easily distinguished from all other species found in France by its coloration pattern (Justine, Gastineau & Winsor, 2024); observed on the ground from breast height, the species displays a clearly visible yellow-orange dorsal band running the entire length of its body. Up close, two dark lines are visible on the yellow band, and on either side of it (Fig. 4). The species has recently been revised (Jones et al., 2020). The main taxonomic result was that *C. bicolor* (Graff, 1899) is now considered a junior synonym. It is also one of the few Geoplanidae species for which we have long-term observations in breeding: one individual kept in breeding was able to live up to five years (Jones et al., 2020).

Individuals found in Europe are all asexual and reproduce by detaching a 10–30 mm fragment from the posterior end of the body, which then regenerates the head organs and a pharynx (Jones et al., 2020). We note that this asexual reproduction is an advantage for an invasive species, since a single individual arriving in a new territory will be able to reproduce alone without having to find a sexual partner. This is particularly the case for a flatworm transported by an animal and deposited in a location where it will be alone.

*Caenoplana variegata* is an arthropod predator. These soil arthropods include isopod crustaceans (woodlice) and insects, myriapods, and spiders. Images of this soft and seemingly fragile worm defeating a large spider are particularly impressive (Fig. 4F in Jones et al., 2020), and citizen science observations in France, not shown). Clearly, this ability to ensnare and kill prey, even those with hard, spine-lined integuments, is due to its particularly sticky mucus. This sticky mucus is responsible for the worm’s adhesion to mammalian fur and makes *C. variegata* particularly well-suited for transport by domestic pets. By comparison, *O. nungara*, a predator of molluscs and earthworms, has much less sticky mucus, as evidenced by the lack of records on domestic animals (Table 2).

### Cats and dogs and their journeys

It is well known that domestic cats and dogs like to go out, either alone for the former, or with their owners for the latter. Data exist for the length of daily journeys taken by domestic pets, especially since GPS collars are available. Of course, there is considerable variation based on size, age, and breed. For dogs, it is estimated that owners walk their dogs for about 30 min per day, or about 2 km (Ham & Epping, 2006; Westgarth, Christley & Christian, 2014). For cats, mean journeys of 2.75 km/day have been measured (Jensen et al., 2022), with mean distances from home ranging 352–588 m (Bischof et al., 2022; Simmons et al., 2023). We used daily journey minimalist estimates of 1 km for dogs and 0.5 km for cats, taking in account that about half of them do not leave their home. When these factors are applied to the number of dogs and cats in France (ca. 10 million and 16 million, respectively) (FACCO, 2024), this leads to an impressive total, in terms of annual distance travelled, of 10 billion km for dogs and 8 billion km for cats, respectively.

The total (minimalist) estimate of the number of kilometres travelled by cats and dogs in a year in a single country, France, is therefore around 18 billion km. This is an astronomical number, representing more than a hundred times the Earth–Sun distance. If only a very small portion of these journeys involve animals with land flatworms attached to them, the possibility of flatworms being efficiently transmitted from garden to garden by animal transport becomes entirely plausible. For the above calculations, to be consistent with our work on data from France, we only considered dogs and cats in this country, but it is clear that this could apply to all countries in Europe or other continents where the worm is present, with millions of dogs and cats, and hundreds of billions of kilometres travelled.

### Other countries, other worms, and other carrier animals

We searched the literature for similar observations and found very few. For the New Zealand flatworm *Arthurdendyus triangulatus* (Dendy, 1895) Jones, 1999 in Northern Ireland, there is mention of four cases of worms stuck to domestic pets, and one on a cow’s hoof, for a total of 1,019 observations (Moore, Dynes & Murchie, 1998), *i.e.,* a percentage of 0.45% of observations related to transport by animals. This percentage is lower than our result for *C. variegata* (7.3%), possibly because *A. triangulatus* has less sticky mucus.

Besides domestic mammals, birds constitute an important part of garden fauna in France and elsewhere. A recent observation in the scientific literature is striking: that of an adult budgerigar (*Melopsittacus undulatus*) with a worm attached to it, which happens to be a specimen of *C. variegata* (Glaw et al., 2024). This observation of a caged animal is obviously not significant, and it is difficult to imagine how a bird could roll on the ground, as dogs do, and then have a terrestrial worm attached to its plumage. However, there are classical observations in the literature of land flatworms climbing tree trunks (Froehlich, 1955) especially for *Caenoplana* species (Moseley, 1877), and therefore the probability of an arboreal bird coming into contact with a land flatworm is not nil. The possibility of transport of land flatworms by birds should be kept in mind, but in the absence of field observations, we cannot estimate if this is significant compared to dogs and cats.

We also aimed to determine whether observations similar to those obtained through citizen science in France were available for the rest of the world. A search on iNaturalist for the taxon Geoplanidae and the keywords “cat” and “dog” yielded 21 results (Table 3). Two of these results concerned *C. variegata*, both in Australia, the worm’s country of origin. The photograph of one record is shown in Fig. 3. Interestingly, observations in the United States (USA) do not concern *C. variegata* (which is not present in this country), but five observations relate to the wandering broadhead planarian, *Bipalium adventitium* Hyman, 1943, a species native to Asia but known only from the northern USA and Canada (Ducey & Noce, 1998; Justine et al., 2019). In Australia, another observation involved a species of the same genus as *C. variegata*, *C. coerulea* (Moseley, 1877), which is also a species with sticky mucus that consumes arthropods. Geographically, these observations were in the USA (14), Australia (5), Brazil (1), and New Zealand (1). As usual, biases inherent in iNaturalist observations are present, in particular the overrepresentation of data from countries where people have smartphones, easy access to the internet, and an interest in nature. However, these observations in iNaturalist suggest that what we found in France has a broader scope than just the species *C. variegata*. It would interesting to explore whether the success of the *B. adventitium* invasion of the USA could also be due, in part, to its ability to be transported by animals.

**Table 3:** Observations of Geoplanidae in iNaturalist. Observations mentioning “cat” or “dog” after removal of irrelevant data (such as worm under dog’s bowl, or locality with “cat” or “dog”). Compiled February 2025.

#	iNaturalist link	Comments by observer	Species	Place
**01**	https://www.inaturalist.org/observations/51056859	“This was a worm-like creature found on my cat’s paw, stuck to some mulch”	Bipaliinae	Texas, USA
**02**	https://www.inaturalist.org/observations/255504985	“Was on my dog which upset her greatly”	*Bipalium adventitium*	Oregon, USA
**03**	https://www.inaturalist.org/observations/143831838	“This fell off of my dog’s paw fur when she came in (it is raining)”	*Bipalium adventitium*	Massachusetts, USA
**04**	https://www.inaturalist.org/observations/111284988	“I was finding these stuck to my dogs legs after they went exploring in my boggy woods”	*Bipalium adventitium*	Vermont, USA
**05**	https://www.inaturalist.org/observations/40288087	“Found on my dog’s paw”	*Bipalium adventitium*	Ohio, USA
**06**	https://www.inaturalist.org/observations/34915718	“Found on the floor of my kitchen. […] Don’t know if it came off my shoe or out of my dogs butt”	*Bipalium adventitium*	Connecticut, USA
**07**	https://www.inaturalist.org/observations/245310668	“Found it in the leg fur of my dog who seemed agitated”	*Bipalium kewense*	South Carolina, USA
**08**	https://www.inaturalist.org/observations/156384559	“Attached to the dogs leg, dog not happy at all”	*Bipalium kewense*	North Island, New Zealand
**09**	https://www.inaturalist.org/observations/65652307	“Hitched a ride into the house on my dog, after a day of heavy rain”	*Bipalium kewense* (?)	Georgia, USA
**10**	https://www.inaturalist.org/observations/138676631	“Found indoors next to the dog”	*Bipalium kewense* (LW)	North Carolina, USA
**11**	https://www.inaturalist.org/observations/236452797	“Pulled it off my small terrier dogs leg (dew claw area)”	*Caenoplana coerulea*	Victoria, Australia
**12**	https://www.inaturalist.org/observations/108007283	“These get stuck in my dog’s hair and seem to irritate him”	*Australopacifica dendyi*	Victoria, Australia
**13**	https://www.inaturalist.org/observations/109693279	“Found on my dog’s butt”	*Caenoplana variegata*	New South Wales, Australia
**14**	https://www.inaturalist.org/observations/145872237	“I found it stuck to my dog”	*Caenoplana variegata*	Australian Capital Territory , Australia,
**15**	https://www.inaturalist.org/observations/63643085	“It came off my dog”	*Dolichoplana striata* (LW)	Mississippi, USA
**16**	https://www.inaturalist.org/observations/256856832	“[…] It was on my dog when we got home from an evening walk - not sure exactly where it came from within the park”	Geoplaninae	California, USA
**17**	https://www.inaturalist.org/observations/127281306	“Found it sucking on my dog, I thought it was some kind of bird feces when I took it out”	Geoplanidae	Brazil
**18**	https://www.inaturalist.org/observations/185359299	“Crawled up my dogs leg”	*Platydemus manokwari*	Florida, USA
**19**	https://www.inaturalist.org/observations/139843327	“Think it fell off our dog or its rope chew toy”	Geoplanidae (LW)	Queensland, Australia
**20**	https://www.inaturalist.org/observations/92420862	“My dog picked it off of her foot after going for a night time walk”	Unidentified Geoplanidae	Pennsylvania, USA
**21**	https://www.inaturalist.org/observations/54352682	“Found on dog that went into backyard briefly”	*Platydemus manokwari*	Florida, USA

### Significance of the transport of terrestrial flatworms by animals

The transport of plant seeds by animals is a well-known phenomenon called zoochory, or more precisely, epizoochory: some plants have developed particularly sticky or spiny seeds or fruits, that attach to animals and are deposited further afield (Lambers, 2025). Some small animals also use this process, particularly small arthropods that attach to a larger arthropod to be transported. Classical cases are ectoparasitic lice transported by hippoboscid flies, free-living nematodes transported by sepsid flies, and mites carried by insects (Campbell, 2002; Combes, 2020; Durden, 2019; Lewis, Campbell & Sukhdeo, 2002). The transport of animals by animals of another, larger species, where the smaller animal is simply transported without any trophic link, is called phoresy (Campbell, 2002; Goater, Goater & Esch, 2014). Phoresy is one of the possible aspects of commensalism, which represents a broad spectrum of interactions between two species (Combes, 2020). One of the extremes of this relationship between two species is parasitism, where one species (the smaller one) feeds at the expense of the other. The presence of flatworms on the integument of vertebrates can be mistakenly compared to the presence of a parasite on the skin. In this case, the word generally used is pseudoparasitism, “pseudo-” indicating that the supposed parasite, namely the land flatworm, does not extract any food from the animal carrying it and should in no case be considered a parasite (Goater, Goater & Esch, 2014). We consider that the temporary transport of land flatworms on the fur of animals is not parasitism (although it could be called pseudoparasitism), but this is a case of short-term phoresy.

Importantly, the presence of land flatworms in the digestive tract of animals or humans is a completely different case from the phoresy mentioned above; in this case, the term pseudoparasitism is justified, since the geoplanid, which is not a parasite, is found in a habitat typical of parasites. A few rare cases have been reported in the literature (Walton & Yokogawa, 1972; Winsor, 1980; Winsor, 1983).

### Veterinary implications

As explained above, cases of land flatworm carriage on animal fur should be interpreted as short-term phoresy and are not parasitism, and therefore, in principle, have no implications for veterinarians caring for small pets. However, two observations (#15 in Table 1: “my cat was acting all weird” and #08 in Table 3: “dog not happy at all”) suggest that pets are aware of the presence of these sticky worms and are affected by them. Veterinarians should reassure owners by explaining that these animals are not parasites.

### Conclusion

In this article, we based our hypotheses on the data available in France, probably the richest database on land flatworms based on citizen science (De Waart et al., 2025). Our conclusion is that while approximately ten species of introduced flatworms are present in France, only one species, the two-tone planarian *Caenoplana variegata*, is transported by domestic animals (dogs and cats). This is despite the fact that this species is not the most abundant overall in the country. Although this transport is rare and concerns only a small portion of the flatworm population, the large number of dogs and cats and the enormous number of kilometres travelled by these domestic animals (our estimate of 18 billion km/year) strongly suggest that transport by pets plays a role in the success of invasion by this species. Therefore, in addition to the initial stages of invasion, which are human and mechanised, there may be a final stage, which is non-human and non-mechanised.

Although our conclusions concern metropolitan France, they apply to all countries where *C. variegata* is present. The compilation of observations in iNaturalist suggests that a comparable phenomenon may be found in other countries, for example in Australia for several native species of the genus *Caenoplana*, and in the USA for *B. adventitium*. Additional citizen science data are needed for other countries.

Regarding citizen science, we note that this work was only possible because we obtained data through direct contact (email) with observers, through which observers added personal annotations (Table 1). Without this, we would have only had simplified data on species, location, and date. We strongly recommend that a “free comment field” always be available when software or websites are offered for receiving citizen science observations (as is the case for iNaturalist, see Table 3), and that this free field also be accessible to researchers when the data are made public.

## Additional Information and Declarations

### Data Availability

The following information was supplied regarding data availability: The anonymous reports are available from GBIF at: Inventaire National du Patrimoine Naturel (2019). Inventaires de la faune exotique envahissante-Inventaire sur les vers plats terrestres introduits en France métropolitaine et en outre-mer. UAR PatriNat (OFB-MNHN-CNRS-IRD), Paris. Occurrence dataset https://doi.org/10.15468/p2y2z6 accessed via GBIF.org on 2025-12-10. The reports and data received by email are available in Table 1.

## References

[ref-1] Bischof R, Hansen NR, Nyheim ØS, Kisen A, Prestmoen L, Haugaasen T (2022). Mapping the catscape formed by a population of pet cats with outdoor access. Scientific Reports.

[ref-2] Campbell JF (2002). Entomopathogenic nematode host-search strategies. The behavioural ecology of parasites.

[ref-3] Combes C (2020). The art of being a parasite.

[ref-4] De Waart S (2016). Exotische landplatwormen in Nederland (Platyhelminthes: Tricladida). Nederlandse Faunistische Mededelingen.

[ref-5] De Waart S, Thunnissen N, Sluys R (2021). Exotische landplatwormen in Nederland (Platyhelminthes: Tricladida). Nederlandse Faunistische Mededelingen.

[ref-6] De Waart SA, Vanhove MPM, Justine J-L, Kmentová N (2025). Going Dutch: European distribution of non-native land flatworm species belonging to Geoplaninae and Bipaliinae with focus on the Netherlands. NeoBiota.

[ref-7] Dorigo L, Lago T, Menchetti M, Sluys R (2020). First records of two alien land flatworms (Tricladida, Geoplanidae) from Northeastern Italy. Zootaxa.

[ref-8] Ducey PK, Noce S (1998). Successful invasion of New York State by the terrestrial flatworm, *Bipalium adventitium*. Northeastern Naturalist.

[ref-9] Durden LA, Mullen GR, Durden LA (2019). Lice (Phthiraptera). Medical and veterinary entomology.

[ref-10] European Union (2025). Commission implementing regulation (EU) 2025/1422 of 17 2025 amending implementing regulation (EU) 2016/1141 to update the list of invasive alien species of Union concern. Official Journal of the European Union.

[ref-11] FACCO (2024). Fédération des Fabricants d’Aliments pour Chiens, Chats, Oiseaux et autres animaux familiers. Les chiffres clés de la population animale en France—baromètre FACCO-ODOXA 2024.

[ref-12] Froehlich CG (1955). On the biology of land planarians. Boletim da Faculdade de Filosofia, Ciências e Letras. Boletim da Faculdade de Filosofia, Ciências e Letras. Universidade de São Paulo, Zoologie.

[ref-13] Gastineau R, Lemieux C, Turmel M, Otis C, Boyle B, Coulis M, Gouraud C, Boag B, Murchie AK, Winsor L, Justine J-L (2024). The invasive land flatworm *Arthurdendyus triangulatus* has repeated sequences in the mitogenome, extra-long cox2 gene and paralogous nuclear rRNA clusters. Scientific Reports.

[ref-14] Gerlach J (2019). Predation by invasive *Platydemus manokwari* flatworms: a laboratory study. Biological Letters.

[ref-15] Ghezzi D (2024). Prima segnalazione della planaria terricola alloctona *Caenoplana variegata* (Fletcher & Hamilton, 1888) (Platyhelminthes, Tricladida, Geoplanidae), nella Lombardia meridionale. Pianura.

[ref-16] Glaw F, Mass R, Glaw T, Schreiner J (2024). Non-native terrestrial planarian species in Germany and Austria, with first locality records of *Caenoplana variegata* for both countries. Spixiana.

[ref-17] Goater TM, Goater CP, Esch GW (2014). Parasitism: the diversity and ecology of animal parasites.

[ref-18] Ham SA, Epping J (2006). Dog walking and physical activity in the United States. Preventing Chronic Disease.

[ref-19] Haubrock PJ, Turbelin AJ, Cuthbert RN, Novoa A, Taylor NG, Angulo E, Ballesteros-Mejia L, Bodey TW, Capinha C, Diagne C, Essl F, Golivets M, Kirichenko N, Kourantidou M, Leroy B, Renault D, Verbrugge L, Courchamp F (2021). Economic costs of invasive alien species across Europe. NeoBiota.

[ref-20] Houghton M, Terauds A, Shaw J (2022). Rapid range expansion of an invasive flatworm, *Kontikia andersoni*, on sub-Antarctic Macquarie Island. Biological Invasions.

[ref-21] Jensen HA, Meilby H, Nielsen SS, Sandøe P (2022). Movement patterns of roaming companion cats in Denmark—a study based on GPS tracking. Animals.

[ref-22] Jones HD (1978). Observations on the locomotion of two British terrestrial planarians (Platyhelminthes, Tricladida). Journal of Zoology.

[ref-23] Jones HD, Mateos E, Riutort M, Álvarez-Presas M (2020). The identity of the invasive yellow-striped terrestrial planarian found recently in Europe: *Caenoplana variegata* (Fletcher & Hamilton, 1888) or *Caenoplana bicolor* (Graff, 1899)?. Zootaxa.

[ref-24] Justine J-L, Gastineau R, Winsor L (2024). Land flatworms (Geoplanidae) in France and French overseas territories: ten years of research. Zoologia (Curitiba).

[ref-25] Justine J-L, Théry T, Gey D, Winsor L (2019). First record of the invasive land flatworm *Bipalium adventitium* (Platyhelminthes, Geoplanidae) in Canada. Zootaxa.

[ref-26] Justine J-L, Thévenot J, Winsor L (2014). Les sept plathelminthes invasifs introduits en France. Phytoma.

[ref-27] Justine J-L, Winsor L, Gey D, Gros P, Thévenot J (2018). Giant worms *chez moi*! Hammerhead flatworms (Platyhelminthes, Geoplanidae, *Bipalium* spp. *Diversibipalium* spp.) in metropolitan France and overseas French territories. PeerJ.

[ref-28] Justine J-L, Winsor L, Gey D, Gros P, Thévenot J (2020). *Obama chez moi*! The invasion of metropolitan France by the land planarian *Obama nungara* (Platyhelminthes, Geoplanidae). PeerJ.

[ref-29] Lambers H (2025). Seed: agents of dispersal. Encyclopedia Britannica.

[ref-30] Lewis EE, Campbell JF, Sukhdeo MV (2002). The behavioural ecology of parasites.

[ref-31] Moore JP, Dynes C, Murchie AK (1998). Status and public perception of the New Zealand flatworm, *Artioposthia triangulata* (Dendy), in Northern Ireland. Pedobiologia.

[ref-32] Mori E, Giulia M, Panella M, Montagna M, Winsor L, Justine J-L, Menchetti M, Schifani E, Melone B, Mazza G (2022a). Opening Pandora’s box: the invasion of alien flatworms in Italy. Biological Invasions.

[ref-33] Mori E, Magoga G, Mazza G (2022b). New records based on citizen-science report alien land planarians in the three remaining Italian regions and Pantelleria island, and first record of *Dolichoplana striata* (Platyhelminthes Tricladida Contineticola Geoplanidae) in Italy. Redia.

[ref-34] Mori E, Touloupakis E, Viviano A, Mazza G (2023). Opening a gate to shade some light: alien land planarians in the Eastern Mediterranean and Northern Africa. Zootaxa.

[ref-35] Moseley H (1877). Notes on the structure of several forms of land planarians, with a description of two new genera and several new species, and a list of all species at present known. Quarterly Journal of Microscospical Science.

[ref-36] Muniappan R (1987). Biological control of the giant African snail, *Achatina fulica* Bowdich, in the Maldives. FAO Plant Protection Bulletin.

[ref-37] Noël S, Fourcade Y, Roy V, Bonnet G, Dupont L (2025). Population dynamics of the exotic flatworm *Obama nungara* in an invaded garden. Ecology and Evolution.

[ref-38] Pantin CFA (1950). Locomotion in British terrestrial nemertines and planarians: with a discussion on the identity of *Rhynchodemus bilineatus* (Mecznikow) in Britain, and on the name *Fasciola terrestris* O F Müller. Proceedings of the Linnean Society of London.

[ref-39] Simmons RE, Seymour CL, George ST, Peters K, Morling F, O’Riain MJ (2023). Seasonal movement patterns of urban domestic cats living on the edge in an African City. Animals.

[ref-40] Sluys R (2016). Invasion of the flatworms. American Scientist.

[ref-41] Vardinoyannis K, Alexandrakis G (2019). First record of the land planarian *Caenoplana bicolor* (Graff, 1899) (Platyhelminthes, Tricladida, Continenticola) in Greece. BioInvasions Records.

[ref-42] Walton BC, Yokogawa M (1972). Terrestrial turbellarians (Tricladida: Bipaliidae) as pseudoparasites of man. Journal of Parasitology.

[ref-43] Westgarth C, Christley RM, Christian HE (2014). How might we increase physical activity through dog walking?: A comprehensive review of dog walking correlates. International Journal of Behavioral Nutrition and Physical Activity.

[ref-44] Winsor L (1980). Pseudoparasitism of domestic and native animals by geoplanid land planarians. Australian Veterinary Journal.

[ref-45] Winsor L (1983). Vomiting of land planarians (Turbellaria: Tricladida: Terricola) ingested by cats. Australian Veterinary Journal.

[ref-46] Winsor L (1990). Taxonomic studies on free-living flatworms (Turbellaria: Platyhelminthes) of the Australian Zoogeographic Region. Chapter 4: taxonomy and biology of a molluscivorous terrestrial flatworm *Platydemus manokwari* Beauchamp, 1962. MSc thesis.

